# Supplementary material for: A Protein–Protein Interaction Analysis Suggests a Wide Range of New Functions for the p21-Activated Kinase (PAK) Ste20
Source: Int J Mol Sci. 2023 Nov 2;24(21):15916. doi: 10.3390/ijms242115916 (PMC10647699; doi:10.3390/ijms242115916)
Supplement: Supplementary file 1 [file ijms-24-15916-s001.zip › ijms-2649271-supplementary.pdf]

Table S1. Proteins identified in the Rdi1 split-ubiquitin screen. Data are from the *Saccharomyces* Genome Database (https://www.yeastgenome.org).

Protein	Molecular function and/or biological process
Adh1	Alcohol dehydrogenase
Aim21	Involved in mitochondrial movement along actin filaments
Apq12	Nuclear envelope protein involved in lipid homeostasis and nuclear envelope organization
Atg8	Ubiquitin-like protein with a role in membrane fusion and autophagosome formation
Bna4	Kynurenine 3-monooxygenase involved in de novo NAD biosynthesis from tryptophan
Bud22	Protein required for rRNA maturation and ribosomal subunit biogenesis
Cdc60	Leucine-tRNA ligase required for synthesis of leucyl-tRNA
Cff1	Protein required for production of 4-hydroxy-5-methylfuran-3(2H)-one
Chs5	Component of the exomer complex involved in golgi to plasma membrane transport
Cns1	Co-chaperone that binds Hsp90 and Hsp70 proteins
Ctf4	Involved in mitotic sister chromatid cohesion and initiation of DNA replication
Cyc8	General transcriptional co-repressor
Ddr48	DNA damage-responsive protein
Emc1	Subunit of endoplasmic reticulum membrane protein complex involved in protein folding
Flc2	FAD transmembrane transporter involved in hypotonic response and calcium homeostasis
Hpc2	Subunit of the HIR nucleosome assembly complex involved in histone gene transcription
Lsb6	1-Phosphatidylinositol 4-kinase
Lso2	Involved in early translational elongation after recovery from stationary phase
Mdm1	Involved in mitochondrial and nuclear inheritance
Mes1	Cytoplasmic methionyl-tRNA synthetase
Met3	ATP sulfurylase involved in sulfate assimilation and sulfur amino acid metabolism
Mhp1	Microtubule-associated protein involved in microtubule organization
Mlc1	Myosin light chain involved in formation and contraction of actomyosin ring
Nop13	Nucleolar protein found in preribosomal complexes
Pai3	Endopeptidase inhibitor involved in vacuolar protein catabolism
Pep7	Adaptor protein involved in vesicle-mediated vacuolar protein sorting
Pop8	Involved in tRNA and snoRNA processing
Rad18	Subunit of the Rad6-Rad18 ubiquitin ligase complex involved in postreplication DNA repair
Rba50	Protein involved in transcription by RNA polymerase II
Rgc1	Putative regulator of glycerol transport by the Fps1p channel
Rsc8	Component of the RSC chromatin remodeling complex
Rsc30	Component of the RSC chromatin remodeling complex
San1	Ubiquitin-protein ligase with a role in degradation of aberrant nuclear proteins
Sdo1	Guanyl-nucleotide exchange factor that contributes to mature ribosome assembly
Sec6	Mediates the targeting and tethering of post-Golgi secretory vesicles to sites of exocytosis
Sec27	Subunit of the COPI vesicle coat complex required for ER-to-Golgi vesicle transport
Sec53	Phosphomannomutase involved in protein targeting to endoplasmic reticulum
Sen1	ATP-dependent 5' to 3' RNA/DNA and DNA helicase
Srb5	Subunit of the RNA polymerase II mediator complex
Srp101	Signal recognition particle receptor alpha subunit
Tea1	Ty1 enhancer activator involved in Ty enhancer-mediated transcription
Tfg2	Protein involved in both transcription initiation and elongation of RNA polymerase II
Tif4632	Translation initiation factor involved in translation initiation and stress granule assembly
Trk2	Potassium ion transmembrane transporter involved in cellular potassium ion homeostasis
Twf1	Involved in actin filament organization and actin cortical patch organization
Wrs1	Cytoplasmic tryptophanyl-tRNA synthetase
Yak1	Protein kinase and component of a glucose-sensing system that inhibits growth

Yat2	Cytosolic carnitine O-acetyltransferase involved in metabolism of alcohol and carnitine
Ydr239c	Protein of unknown function
Yih1	Protein kinase inhibitor that binds actin monomers, ribosomes, and polysomes
